# Patients’ expectations to dental implant: a systematic review of the literature

**DOI:** 10.1186/s12955-014-0153-9

**Published:** 2014-10-29

**Authors:** Jie Yao, Hua Tang, Xiao-Li Gao, Colman McGrath, Nikos Mattheos

**Affiliations:** Faculty of Dentistry, the University of Hong Kong, 34 Hospital Road, Sai Ying Pun, Hong Kong, SAR PR China; Department of Oral Implant Center, West China Hospital of Stomatology, Sichuan University, Chengdu, China

**Keywords:** Dental implant, Patients’ expectations, Systematic review

## Abstract

**Objective:**

To examine the current literature on the impact of patients’ expectations on treatment outcomes or final patient satisfaction and to identify the theoretical frameworks, study designs and measurement instruments which have been employed to assess patients’ expectations within implant dentistry.

**Methods:**

A structured literature search of four databases Pubmed, Cochrane, Web of Science and PsychINFO was conducted following PRISMA guidelines. Any type of literature published in English discussing the topic of ‘patients expectations’ in oral health were identified and further screened. Studies reporting on expectations regarding dental implants were selected and a narrative review was conducted.

**Results:**

The initial search yielded 16707 studies, out of which 1051 ‘potentially effective studies’ were further assessed and final 41 ‘effective studies’ were included [Kappa = 0.76]. Ten observational studies, published from 1999 to 2013, dealt specifically with expectations of dental implants. There was a large degree of heterogeneity among studies in terms of assessment instruments. Expectations relating to aesthetics and function were primarily considered. Among the 10 studies, 8 were classified as quantitative research and 2 as qualitative research. The STROBE quality of reporting scores of the studies ranged from 13.5 to 18.0. Three of the 8 quantitative studies employed a before/after study design (prospective studies) and used visual analogue scales (VAS) to measure patient expectations.

**Conclusions:**

There is a growing interest in patients’ expectations of dental implants. Most studies are cross sectional in nature and the quality of reporting varies considerably. Expectations with respect to aesthetics and function are key attributes considered. The use of visual analogue scales (VAS) provides quantitative assessments of patients’ expectations but the lack of standardization of measures prohibits meta- analyses.

## Introduction

Quality assurance of health care delivery has emphasized in the importance of patient’s perceptions of medical interventions and treatments since 1970s [1]. The view that patient expectations from a treatment play a potential role to their final satisfaction from the treatment outcomes has intrigued clinicians and researchers [2]. This is even more critical today, as the current practice of Evidence Based Medicine requires that the patients are actively engaged in the decision making with regards to their treatment. In addition, understanding and measuring the expectations of patients prior to treatment appears to be an essential prerequisite to achieve successful patient reported clinical outcomes.

Broadly speaking, expectations are beliefs about future consequences, which may contribute to an individual’s psychological and physiological change [3]. In medicine, the variety appears in the concept, type and usage as well. According to the literature review published in 2012 by Ann Bowling and coworkers [4], the current literature failed to address the multidimensionality of this concept. Moreover, the measurement instruments used to assess expectations are very diverse, without validity and reliability test. Thus, there is a strong need to further develop the concepts of patient expectation and investigate both theoretically and empirically its implications for patient reported treatment outcomes. This need is even more pronounced with regards to treatments with dental implants, where expensive therapy is proposed for the rehabilitation of function and esthetics of patients with missing teeth. Satisfaction after treatment with dental implants appears to be evident in a number of studies. According to a prospective cohort study of patients’ satisfaction following implant therapy in 10 years, more than 90% of the patients were completely satisfied with implant therapy [5]. Nonetheless, as one of the relatively new technics in dentistry, implants are still unknown to a wide segment of the population. Saha and coworkers conducted a survey in 2013 among 483 subjects to assess the awareness regarding implants and the authors indicated that more than half of the participants had no information of implants [6]. This conclusion is consistent with other studies published in recent years [7-9]. The lack of reliable information may be one reason leading to the development of patients’ unrealistic expectations. Another possible resource for unrealistic expectations is the perceived “novelty” of this treatment, especially when coupled with the high cost of the implant therapy. Based on the view that patients’ unmet expectations would negatively influence their satisfaction with the treatment outcome, identifying patients’ expectations before the treatment is a necessary step to prevent patient disappointment with the final treatment outcomes.

A systematic review of the literature was conducted aiming to review available evidence with regards to patients’ expectations from clinical treatments within comprehensive oral healthcare. The aim of this paper is to report the literature review outcomes within the discipline of implant dentistry. In particular, this study aims to review the evidence with regards to:

- impact of patients’ expectations on treatment outcomes or final patient satisfaction with treatment outcomes within implant dentistry
- theoretical frameworks, study designs and measurement instruments which have been employed to assess patients’ expectations within implant dentistry.

## Methods

### Study protocol and eligibility criteria

A wider “umbrella” search protocol was developed in order to identify evidence on the impact of patients expectations in outcomes of oral healthcare. Two independent researchers conducted the search. Studies were initially included if they met the following criteria:

1. Human subjects were investigated with regards to their expectations from dental treatment.
2. Experimental studies (randomized or not, prospective, retrospective and cross sectional) with qualitative and/or quantitative analysis.

### Search strategy and data resources

Since patient “expectations” represent a rather new area in dental research, no suitable MeSH term was available. A search was broadly employed to identify as many relevant studies as possible. The overall search strategy was defined for comprehensive oral health, thus used the text words ”expectation” and MeSH terms “knowledge”, “attitude”, “oral”, “dental”, “dentistry”. All papers found reporting within oral healthcare were further organized in dental disciplines. The studies reporting on dental implants were selected for further analysis in this paper. An additional specific search was conducted with the keyword “expectation” and the MeSH term “dental implants”, which however didn’t add any further papers (Table 1).

**Table 1:** **Search strategy**

#1 = expectation	#13 = attitude
#2 = patient expectation	#14 = patient attitude
#3 = expectation satisfaction	#15 = attitude belief
#4 = health expectation	#16 = attitude scale
#5 = treatment expectation	#17 = attitude knowledge
#6 = expectation outcome	#18 = attitude questionnaire
#7 = patient expectations satisfaction	
#8 = expectant	
#9 = expected value	
#10 = expected	
#11 = outcome expectations	
#12 = #1 OR #2 OR #3 OR #4 OR #5 OR #6 OR #7 OR #8 OR #9 OR #10 OR #11	#19 = #13 OR #14 OR #15 OR #16 OR #17 OR #18
#20 = knowledge	#28 = oral
#21 = knowledge attitude practice	#29 = oral health
#22 = health knowledge	#30 = oral care
#23 = knowledge questionnaire	#31 = dental
#24 = patient knowledge	#32 = dental health
#25 = knowledge practice	#33 = dental care
#26 = oral health knowledge	#34 = dentistry
#27 = #20 OR #21 OR #22 OR #23 OR #24 OR #25 OR #26	#35 = #28 OR #29 OR #30 OR #31 OR #32 OR #33 OR #34
#36 = #12 AND #35	**Specific search**
#37 = #19 AND #35	#39 = dental implants [MeSH]
#38 = #27 AND #35	#40 = #12 AND #39

**Figure 1:**
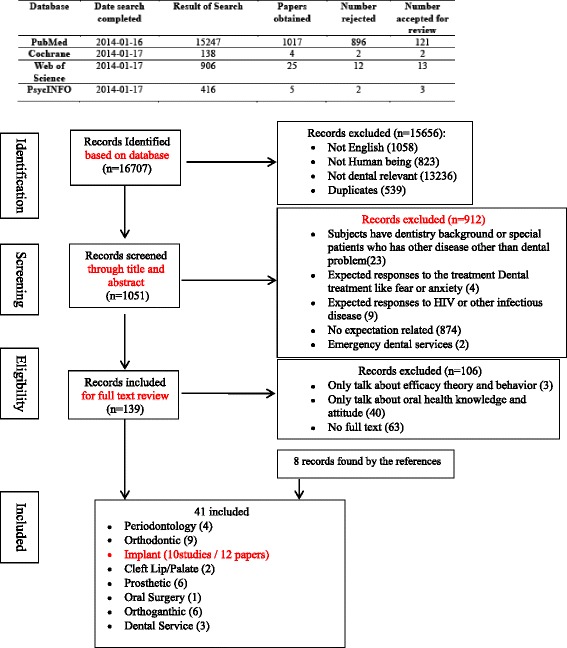
**Phases in the development of eligible literatures.**

Literature search results originated from the online databases: Pubmed, Cochrane, Web of Science and PsychINFO. No starting point was set in time and the final search was run on 19 September 2014. Any type of literature with the patients’ expectation topic in oral health was included to the initialy screened and the hand search extended to the references listed in the included studies [Figure 1].

### Study screening and data extraction

Two reviewers (JY and HT) screened the title and abstract of each citation independently to determine whether the study would be further retrieved in full text. Based on the pre-determined eligibility criteria, studies with a clear description of the aim, method (e.g. sample type and size, study design) and result were considered. Full-text of the possible eligible studies were retrieved. After the assessment of the full text, decision was made by the two reviewers for final selection. The inter-reviewer agreement for each eligibility citation was calculated as described by Cohen J. [10]. Disagreements were resolved by discussion in the series of stages. In case of disagreement, other co-authors were involved in discussion until consensus was reached.

Once the satuies were selected for final analysis, the following data of each study was extracted by one reviewer (JY): author, year of publication, name of journal, subjects (age, diagnosis, and previous prosthodontic experiences), study design, measurements (instrument, questionnaire items and interview topic) and results. The second reviewer (HT) controlled the extracted data and if any objection or disagreement occurred, this was resolved by consensus. Meta-analysis of the results was not possible due to the wide range of study designs and sample types. Thus a narrative synthesis was undertaken.

### Analysis and quality assessment

The criteria in *Strengthening the Reporting of Observational Studies in Epidemiology (STROBE)* were utilised to evaluate the study quality [11]. The *STROBE* statements represent the quality standards of observational studies (cohort, case–control and cross-sectional studies). The 22 items in *STROBE* provided guidance to assess the title, abstract, introduction, methods, results and discussion sections. Two investigators rated the score for each study (fully met = 1; Partial met = 0.5; N/A or Not at all = 0). The mean scores of two raters were recorded as the final quality score.

## Results

### Study selection

The search of four databases (Pubmed, Cochrane library, Web of Science and PsycINFO) initially provided a total of 16.707 citations. The earliest paper was published in 1966 and was available in Pubmed. After adjusting for duplicates (539 studies), language (1058 studies) and subjects (823 studies), 13.236 studies were further excluded because of no relevance to oral health. Of the remaining 1051 articles, the second round screening discarded 912 studies through evaluating the abstracts. Studies were excluded because of:

1. Not investigating an expectation/anticipation/request/need in the study aims (874 studies)
2. Investigating response expectations to the treatment like fear or anxiety (4 studies) Response expectations are investigated in systematic desensitization therapy and they are anticipations of automatic reactions to particular situational cues [12].
3. Investigating response expectations to HIV or other infectious disease in dental treatment (9 studies)
4. The study sample having dental background or special diseases other than involving dental problems (23 studies)

The full texts of remaining 76 studies were examined in detail. Forty-three studies were excluded during the final round screening because of insufficient research approach to the investigation of expectations. For example, some papers mainly investigated “self-efficacy” which we think is only a sub-determiner of expectations [13]. In addition, 8 studies were chosen from references by hand search [14-21]. Thus, a total of 41 studies investigating patients’ expectations in oral health were identified in the final analysis. The eligibility criteria were consistent during all the stages of screening and the mean kappa value for the agreement between the reviewers was 0.76. Of these 41 studies, 10 studies (12 papers) published from 1999 to 2013 were identified to measure expectations of dental implants and were thus further analyzed for the purpose of this paper. In addition, the specific search strategy “expectation” combined with “dental implants” did not offer any new eligible papers.

### Study characteristics

Out of the 10 implant related studies (12 papers), 8 were quantitative research (10 papers) and 2 were qualitative research (2 papers). The sample size ranged from 9 to 1000 subjects. The age range was not clear because some papers just provided the mean age. The study countries were UK for two studies, Austria for three studies, Brazil for two studies, Sweden, Canada and Germany for one study, respectively. Implant treatment included implants supported single crowns, fixed partial dentures and over-dentures (Table 2). All 10 studies (12 papers) were observational studies with the *STROBE* score ranging from 13.5 to 18 (total score = 22). Based on the content in *STROBE*, the highest score (≧9) are rated for the *title, abstract, introduction (background and objectives), study design, outcome data and discussion (key results, interpretation, generalizability).* The lowest score (≦2.5) are rated for *bias description, study size explanation, and limitation discussion.*

**Table 2:** **Study Characteristics of analyzed studies**

**Title**	**SROBE**	**Sample size**	**Sample character**	**Design**	**Method**	**Study character**	**Result**
Baracat, 2011 [22]	13.5	50(mean age 49, SD 11.45)	• 50 patients seeking dental implant treatment	Before/after	VAS	• VAS to assess patients expectations (esthetic and functional (mastication, comfort, retention results) before and evaluation after(one week) implant treatment	• The post-treatment completion ratings significantly exceeded expectations
			• The prosthetic procedures ranged from single crowns to full fixed bridges, covering fixed partial bridges and overdentures on two ball abutments.				• Positive correlations were found between expectations and posttreatment completion ratings for esthetics
							• An inverse correlation was found between age and functional expectations
Heydecke, 2008 [23]	15.5	162 (102, middle aged, ages 35 to 65, MEAN = 51.1, SD = 7.5. 60 senior, ages 65–75, MEAN = 69.3, SD = 3.1)	• Participants included in are edentulous for at least 5 years.	Before/after	VAS	• Before randomization, each subject rated their satisfaction with their	• Post treatment satisfaction with CD treatment was significantly lower than pretreatment expected satisfaction in both study populations (MA, P < .0001; Senior, P = .036).
						current denture and expectations of satisfaction with both IOD and CD treatment	
			• Both group receive a maxillary conventional denture, then separate into two group				
						• 6 months post-treatment, all rated	• There was no (or only borderline) significant difference between pretreatment expectation and posttreatment satisfaction for patients receiving IODs in both study populations (MA, P = .078; S, P = .057).
						their satisfaction with their new prostheses on similar VAS.	
			• IOD: 2-implant-supported overdenture				
						• Expectations and satisfaction with treatment were compared.	
			• CD: conversional overdenture				
de Lima 2012 [24]	17	52(28–77 years; Mean = 51.2; SD = 10.6)	• 52 individuals who had received implant therapy	Before /after	VAS	• VAS was used to assess expectation before and satisfaction after therapy	• Patient expectations before treatment were higher than satisfaction after treatment, but this difference was significant only for esthetics in patients who had received implant-supported FPDs.
						• After treatment, Patient also assigned scores for four aspects before and after treatment: mastication, aesthetics, phonetics and comfort of use.	
			• These individuals received implant-supported single crowns or FPDs (all metal-ceramic) using a standardized technique.				
						• Likert type scale to gauge patient evaluations of clinician conduct based on previously developed questionnaires.	
			• If patients received both treatment options, they were included in the FPD group for analysis.				
							• Negative correlations were found between satisfaction and age and between number of absent teeth and number of post-delivery adjustments, but only for implant-supported FPDs.
							• A positive relationship was found for the majority of questions concerning patients’ evaluations of clinician conduct and VAS scores.
Hof, 2012 [25]	15	150(Age 18–84 years)	• 150 consecutive patients seeking implant treatment	Cross-sectional	Survey/Scale	Questionnaire One: rank their concerns regarding implant therapy by priority	• Patient expectations on implant success and predictability are high compared with their reluctance towards treatment costs and duration.
			• One quarter of patients wore removable dentures			• predictability of treatment success	• Acceptance of treatment morbidity is high among patients reporting low denture satisfaction
						• time	
			• 40% had fixed restorations			• cost efficiency of treatment	• Minimally invasive treatment alternatives are generally preferred.
						• avoidance of removable dentures or bone grafting	
			• 9%edentulous				
						Questionnaire Two: 16 questions	
						• Expectation items	
						• Acceptance and Preferences items	
						• Second-opinion seeking items	
I/II: Pommer 2011 [8,26]	18	1000	• 1000 adults over 14 years of age representative for the Austrian population	Cross-sectional	Survey	A. 19 items questionnaire	• Compared with the survey of 2003, the subjective level of patient information about implant dentistry has significantly increased in the Austrian population.
						• Implant Information items	
						• Sources of information	
						• Need for patient information about dental implants	
			• Sample was selected by pre-stratified multi-tiered cluster sampling using factor weighting for the variables sex, age, level of education, net monthly income and size of residence.				• The objective level of general knowledge about dental implants was still all but satisfactory revealing unrealistic patient expectations.
							• dentists are still the main source of patient information
						B. Same with Tepper 2003 [27,28]	• The majority felt that only specialists should perform implant dentistry.
I/II. Tepper, 2003 [27,28]	13.5	1000	• 1000 adults over 14 years of age representative for the Austrian population	Cross-sectional	Survey	B.16 items questionnaire	• Of those familiar with implants as one of the treatment alternatives, 61% reported they would accept implants if the need arose.
						• Dental implant acceptance	
						• Perceived Costs	
						• Patient satisfaction	
						A. Same with Pommer.2011 [26]	
			• Random sampling				• Implant acceptance was highest among males and interviewees below the age of 30 years.
							• The interest in implants increased with increasing family incomes.
							• All those questioned found implant-supported rehabilitation to be very expensive.
							• Many of them blamed the dentists for the high cost.
							• One detail was particularly evident: satisfaction among implanted patients was clearly higher than satisfaction rates perceived by them from what they were told about implants by others. First-hand experiences with implants proved to be less biased than reported second-hand information.
Rustemeyer, 2007 [9]	15	315	• mean age 55 +/−14.7 years	Cross-sectional	Survey	7 special questions about implants to gauge the patient’s perceptions of oral hygiene considerations, durability and costs of an implant-supported overdenture, as well as the influence of laymen and media in these perceptions.	• 58% of 315 patients questioned thought that implants require the same care as natural teeth,
			• no specific seeking implant treatment patients				• 61% expected an additional payment of 2000 Euro or less,
			• 26 subjects with full dentures, extractions were planned, 121 patients had unsupplied gaps between teeth in the upper or lower jaw, 98 patients had removable dentures with clamps and 71 patients had complete prostheses.				• 80% held the function of an implant-supported overdenture as very important
							• 54% attached great importance to the aesthetics.
							• The expectations that patients have for an implant-supported set are high in contrast to their willingness to make additional payments. There are still misconceptions regarding costs, and these must be resolved individually in practice.
Allen, 1999 [29]	17	61(Implant group:40–83 years old)	This study included two groups:	Case/Control	Scale	• The questionnaire consisted of two scales: (a) a subjective appraisal of the patient existing conventional dentures, and (b) their expectations of an implant-retained prosthesis.	• Baseline satisfaction with current dentures was low in both groups, with the implant group being significantly less satisfied with comfort and stability of their mandibular dentures.
			1.Patients requesting implants to retain a complete prosthesis (implant sample group)				
							• Perceived ability of the implant group to chew hard foods was less than the control group.
			2. A control group of edentulous patients, of similar age and gender distribution as the implant sample group, requesting replacement of their dentures by conventional means.				
						• Variables assessed for existing maxillary and mandibular prostheses were: (1) general satisfaction, (2) satisfaction compared with natural teeth, (3) retention, (4) stability, (5) comfort (6) appearance, (7) the ability to speak, (8) occlusion, and (9) the ability to chew and swallow sliced bread, cheese, carrots, bacon, lettuce, apples and nuts.	
							• The implant group’s expectations of an implant-retained prosthesis were significantly greater than for a conventional denture.
Grey, 2013 [30]	15.5	9 (49–69 years old)	Seven participants had completed implant treatment, one was currently undergoing treatment and one had decided against them.	Cross-sectional	Interview	• Appearance: individual and the social appearance	• The main theme to emerge was ‘normality’. Participants expected implants to restore their oral- related quality of life to ‘normal’.
						• Function	• Patients’ belief that dental implants are just like natural teeth could be cause for concern if it leads them to treat them as such, and thereby not follow the recommended specialist care they require.
Johannsen, 2012 [31]	15.5	17 (46–81 years old)	10 patients who had undergone dental implant treatment. Seven patients who had been treated with dental implants.	Cross-sectional	Interview	All patients in the study had a previous history of periodontal disease with, in most cases, many years of treatment.	• A core category was identified as “Transition from tooth loss, to ‘Amputation’, and to implants – negative and positive trajectories”.
							• Treatment with dental implants improved function, enhanced self-esteem, social life and, thus quality of life.

### Quantitative studies

Three of the 8 quantitative studies utilized a before/after study design with the use of *visual analogue scales (VAS)* to measure the expectation pre-treatment and the actual satisfaction [23,24] or evaluation [22] post-treatment. For example, all three studies asked patients to rate their expectations of the functional and esthetic change brought by dental implants. The items related to function were constructed either as a general idea [22,23] or specific regarding to mastication, phonetic, comfort use and retention issues [24]. Among the three, one study claimed that post-treatment satisfaction ratings significantly exceeded expectations [22]. However, another study reported satisfaction lower than pre-treatment, expectations, especially for esthetics in patients who received implant supported fixed partial dentures [24]. All three papers considered gender, age and placement area as the variables influencing the expectation rating. Baracat and coworkers found negative correlations between age and functional expectations [22]. Heydecke and coworkers concluded high expectations of *IOD (two implants supported over-denture)* treatment were predictive of higher resultant evaluation only in the middle age group (35–65 years old) [23].

In conclusion, seven cross-sectional studies reported in 9 papers employed survey or rating scales (including papers using VAS). Most of studies aimed to assess patients’ knowledge, awareness, expectation, information level and acceptance to dental implant. The sample varied from general population without treatment need [8,9,27], patients who were seeking implants [25] and patients who had completed implant treatment [30,31].

### Qualitative studies

Two qualitative studies interviewed subjects who had completed implant treatment [30,31]. Grey and coworkers revealed participants expected to a “normalization” of their oral-health related quality of life from implant treatments, however, this “normalization” idea was abstract and individual [30]. Both two studies found patients affirmed the improvements brought by implants in their physiological, social and psychological related quality of life [30,31].

### Reported parametres

The parameters investigated in the different questionnaires were very diverse among studies (Table 3). The most frequently used questions were about survival time (5 studies), cost (5 studies), special oral hygiene maintenance (4 studies), information sources (3 studies) and outcome improvements like functional and aesthetical changes (4 studies). For the longevity of dental implants, Hof and coworkers [25] found that 59% of the subjects believed implants could last for a lifetime. However, the same result in Pommers’ [8] was 24% and Teppers’ [27] 34%. Seven percent of the participants in Rustemeryer’s study believed implants could last longer than 25 years [9]. Most current studies pointed out that patients believed the cost of implant treatment to be high. The treatment cost related to income was one of the determinants to hinder subjects from making treatment decisions [9,25,26,28,31]. Three studies assessed the information sources of patients with regards to dental implants and showed the majority of the patients to be informed from the dentist, however to a varying extend of 68% [27], 41% [9] and 74% [8] respectively. Another common question was whether implants need special care. The answers were similar among studies. Less than 6% participants thought dental implant need less oral hygiene care than natural teeth [8]. The data in Rustemeyer’s study was 7% [9] and Tepper’s was 4% [27]. Four studies discussed treatment outcomes (included 2 qualitative studies). Allen and coworkers applied ordinal scale to prove subjects expected dramatic improvement in stability, retention and comfort of implant-retained prosthesis, especially for mandibular (Mean media score = 2.0, very satisfied) [29]. Rustemeyer stated most patients regarded the functional and aesthetics improvements as something important and the percentage of women who judged aesthetical change as vital was significant higher compared with men (68% and 41% respectively, P < 0.05) [9]. In addition, four studies pointed out the unrealistic expectations of implant in patients’ mind [8,9,23,29]. Hof and coworkers still investigated issues related to bone graft in implant surgery and the results showed patients preferred the minimal invasive treatment alternatives [25].

**Table 3:** **Specific Parameters summary in cross sectional studies**

**Parameter**	**Item**	**Main finding**	**Study**	
			**Author,Year**	**N**
Survival time	Fix dentures live longer than removable dentures		Hof, 2012 [25]	5
	Implant supported dentures last longer than toot h supported dentures			
	Predictability of treatment success			
	Dental implants last for a life time	59% the life time	Hof, 2012 [25];	
		24% the life time	Pommer, 2011 [26];	
		34% the life time	Tepper, 2003 [27,28]	
		7% >25 years; 66% :10–20 years	Rustemeyer, 2007 [9]	
		all patients expected the implants to function in the mouth during the rest of their lives	Johannsen, 2012 [31]	
Treatment duration	Healing period of at least 2 months after tooth extraction		Hof, 2012 [25]	1
	Healing period at least 2 months after implant placement			
	Time efficiency of the treatment			
Risks/ Complications	Acceptance of higher risk of failure to shorten your total treatment duration		Hof, 2012 [25]	3
	Who/What would you blame for implant loss?		Pommer, 2011 [26]; Tepper, 2003 [27,28]	
Bone graft Surgery	Avoidance of bone grafting		Hof, 2012 [25]	1
	Acceptance to undergo bone graft surgery to enable dental implant placement			
	Acceptance of extra oral bone graft surgery			
	Acceptance of Intraoral bone graft surgery -chin			
	Acceptance of Intraoral bone graft surgery –retromolar region			
	Acceptance of Bone substitute material			
Cost	Cost efficiency of the treatment	1/3 refuse additional cost; feel cost is barrier	Hof, 2012 [25];	5
		Most patients can not cover the cost of implant treatment	Rustemeyer, 2007 [9]	
		76% feel expensive	Tepper, 2003 [27,28]	
		83% too expensive ; income has relationship to choose implant	Pommer, 2011 [26]	
		The costs were also considered worthwhile even though some of the patients perceived them high	Johannsen, 2012 [31]	
	Acceptance of addition cost of guided implant surgery to avoid bone graft surgery			
Special dental care	Keep Oral hygiene	6% less care then natural teeth	Pommer, 2011 [26];	4
		7% less care then natural teeth	Tepper, 2003 [27,28]	
		4% less care then natural teeth	Rustemeyer, J.2007 [9]	
		The patients perceived that the oral hygiene procedure was too time-consuming with the new teeth, which highlighted another import issue.	Johannsen, A. 2012 [31]	
Second opinion seeking	Would you be content with a removable replacement denture		Pommer, 2011 [26]	3
			Tepper, 2003 [27,28]	
	fixed dentures are not possible without placement of dental Implants		Hof, 2012 [25]	
	Placement of dental Implants is not possible in your specific case			
	Placement of dental implants is not possible without previous bone graft surgery			
	Placement of dental implants is not possible without previous CT			
	Avoidance of removable dentures			
Adequate information	Alternatives of replacing missing teeth	Implant supported	Pommer, 2011 [26]	2
		Removable partial	Tepper, 2003 [27,28]	
		Removable complete		
		Fixed partial		
	Be well informed about implants			
	Be well informed about other restore methods			
	Disadvantage of implant supported dentures	High costs		
		Need of surgery		
		Long treatment time		
	Advantages of fix vs removable	Less annoying in the mouth		
		Look nicer		
		As good as natural teeth in function		
		Do not feel like a foreign body		
	More information is needed			
Information source	Where to get	74% from dentists; Well informed 9%	Pommer, 2011 [26];	3
		68% from dentists; Well informed 4%	Tepper, 2003 [27,28]	
	Where you would like to get	41% from dentists	Rustemeyer, 2007 [9];	
		Friends and acquaintances		
		Media		
Role model	Successful Experiences from friends		Rustemeyer, 2007 [9]	1
GP/specialist	Need Better qualified		Pommer, 2011 [26]	2
	Use up-to-date implant techniques		Tepper, 2003 [27,28]	
Outcome	Aesthetic		Rustemeyer, J.2007 [9]; Allen, 1999 [29]; Baracat, 2011 [22]; de Lima. 2012 [24]; Grey, 2013 [30]; Johannsen, 2012 [31]; Tepper, 2003 [27,28]	7
	Function	Speaking - Phonetics	Allen, 1999 [29]	7
		Occlusion	Baracat, 2011 [22]	
		Mastication/Chew ability	de Lima. 2012 [24]	
		Swallow ability	Johannsen, 2012 [31]	
		General function change	Rustemeyer, 2007 [9]; Heydecke, 2008 [23]; Grey, 2013 [30]	
	Retention		Allen, 1999 [29]	
	Stability		Baracat, 2011 [22]	
	Comfort		de Lima. 2012 [24]	
	Improve quality of life		Johannsen, 2012 [31]	
Satisfaction	Compared with natural teeth		Allen, 1999 [29]	6
	Compared with current prosthesis		Baracat, 2011 [22]	
	General satisfaction		Heydecke, 2008 [23]	
			de Lima. 2012 [24]	
			Tepper, 2003 [27,28]	
			Pommer, 2011 [26]	

## Discussion

To our knowledge, this is the first systematic review on patients’ expectations of dental implants. The area of implant dentistry is a relatively new modality in oral healthcare, which involves rehabilitation treatments with often significant costs. It is also a treatment modality which the patients have little experience and understanding of prior to becoming recipients of implants. Information about implants is widely available through Internet and social media, but with limited quality assurance and often misleading or inappropriate content. As communication bias, uncertainties of diagnosis and therapy often lead to misunderstandings, all unmet expectations may cause future dissatisfactions. For these reasons Implant dentistry was singled out as the focus of this review.

Ten studies reported in 12 papers are characterized by various study designs and sample types. The vague concepts of expectation and the non-standard instruments used among studies provide weak evidence for clinical reference. This prevented any attempt to conduct a meta-analysis. Therefore, the focus of this study is to narratively synthesise the conclusions, as well as evaluate the methodological characteristics of the available studies.

### Main outcomes and evaluation

Expectations of improvements resulting from treatment are the main focus of 10 studies. Seven of these studies [9,22-24,29-31] measured the outcome expectations with simple questions like “Do you expect implants improve the functional and esthetic conditions?” Two studies measured the general functional change after the treatment [22,23]. In another study, instead of specific measurement, patients were asked with regards to chewing ability, phonetic feeling, etc. [24]. Two papers used *visual analogue scales (VAS)* in measuring pre-treatment expectations and post-treatment satisfactions from the outcomes [23,24]. Interestingly, the results were not always positive. Specific items like mastication, phonetic, comfort use and retention issues showed lower satisfaction after treatment [24] than the pre treatment expectations. One of the reasons may be that patients with more detailed considerations of functional experiences may be more sensitive to the change in oral conditions.

The high cost of implants is emphasised in most studies. Patients are reported to often complain about the high cost and many believe this will prohibit them from receiving implant therapy. High cost may also be one of reasons contributing to unrealistic expectations. In our review, 4 papers [8,9,23,29] with big sample size found unrealistic expectations often existed among patients. Although the dentists largely remains the main information resource with regards to implants at present [8,9,27], the reliance of patients is diverse, varying from 41 to 74%.

All studies analyses were observational in terms of design. The definitions and concepts of expectations were simple and one-fold without deeper exploration. Studies seldom discussed the definition of expectations and miss-interchangeable concepts were identified. For example, a lot of studies used the terms “expectations” as synonyms to “need”, “perspectives”, or “requests”, etc. [8,25,27,31]. Actually, these are possibly similar sounding terms in everyday language, but are very diverse when used in scientific terminology within Psychology. The ambiguous definitions can confuse readers when encountering different concept models without well-integrated interpretations. These issues also constitute a major difficulty when scientifically investigating the expectations related topics.

Due to the diversity of definitions (or absence of them) for expectations, the studies included in this review utilised different methodologies and sample types, which also increased the risks of bias. Not surprisingly, the studies identified in this review are weak in bias interpretation. Two qualitative studies investigated expectations of patients who had completed implant treatment [30,31]. These retrospective analyses offer an improved understanding of expectations, as related to simple one-off studies. The relatively longer study period, extending to before and after treatment may guide investigators to gain more detailed insights into patients’ mind. The other sample types consist of patients who are seeking implants [25] and general population without treatment need [8,9,27]. The differences in treatment need may significantly affect the passive or active thinking and patients’ expectations.

Outcome expectations are emphasised. However, as the instruments employed in studies are not always optimal, research results cannot be understood within a consequent context. Research in expectations within the discipline areas related to orthodontics [32] and periodontics [15,16] appear to have instruments with good validity and reliability. The expectations concepts are explored deeper and multidimensional as well. This might be due to the fact that implant therapy is relatively new, when compared with the two other well-established disciplines.

A large body of literature in this review discusses the association between expectations and satisfaction, which is also a hot topic in other fields related to *patient-reported outcomes (PROs*) [33,34]. In contrast to expectations, which are relatively new concept, patient satisfaction has been investigated longer and in more depth. From the systematic review by Crow [2], a census that expectations could predict satisfaction cannot be reached. However, many researchers believe the potential influence of patient expectations and the change of expectations may significantly impact the final satisfaction with a treatment [3,12]. This should be further investigated in future experimental study.

### Limitations and future directions

Any attempt to review evidence in the field of “expectations” in oral healthcare is limited by the lack of a standardized terminology and widely accepted definitions. Consequently, it was a strategic decision of the authors to adopt a “sensitive” rather than a specific search strategy in order to assess as many potentially relevant papers as possible. For this reason, keywords such as “knowledge” and “attitudes” were also included in the search, although not expected directly relevant to the focus area. As it was shown, some papers included relevant data, although they would have not been found by more specific search. For example, Pommer and Teppers’ research on access of patients’ information to dental implant [8,27], also report findings on patients’ expectations. The search strategy this way also provided papers within other dental disciplines, which although are not the focus of this paper, might be reviewed in future studies.

The search of literature is restricted to English-language publications. The search strategy was broad with the aim to find as many relevant studies as possible. Nonetheless, the search process was only limited to electronic databases. Due to the ambiguity in the definitions of expectations and related concepts selection bias is not unlikely, although effort has been taken to minimize it through the methodology and the utilisation of two reviewers. With the heterogeneity in study designs and sample types, the results were extracted with an inevitable degree of subjectivity. Implant Dentistry as evolved tremendously in the last two decades and one can expect that patients attitudes and expectations have also evolved in time. However as one of the aims of this study was to also assess theoretical frameworks and instruments used, it was decided to not set a starting time point for the search. In reality this proved to be not significant, as the few publications available are clustered mainly in the decade 2000–2010.

Expectations should be considered multidimensional and malleable during different clinical stages. Questions using generic ideas/ definitions of expectations may lead to deviation from specific concepts. The patient expectations should be better studied within corresponding scenarios. For example, patients’ expectations from treatments in public hospitals may be significantly different when compared with private clinics. Researchers could better address this when considering of the specific situations the patients may encounter, what kind of expectations they may form and how this would be influenced by sub-determinants such as previous experiences, personal characteristics, social and psychological factors.

In sum, there is a need for future studies designed to:

1. Produce a specific and theoretically sound definition of patients’ expectations from implant treatments, which addresses the complex nature of the phenomenon.
2. Construct the theoretical model of how patients form expectations from dental implant treatment and demonstrate its determinants and contributing factors both theoretically and experimentally.
3. Classify the changes or different roles of expectations at different clinical stages, through a longitudinal study design.
4. Build standardized instruments to help objectively assess patient expectations and better understand how these expectations are formed and developed.
5. Clarify the impact of expectations to the final satisfaction with treatment outcome

## Conclusion

Expectations from dental implants have been investigated in a diversity of approaches within the available literature. The biggest part concerns outcome expectations of improvements in functional and esthetic aspects of treatment. The current findings of research are limited by weak study design and non-standardized instruments which decrease the level of evidence. Unrealistic expectations are often found among patients, which may lead to dissatisfaction with final outcomes. The concept of expectations should be further developed theoretically and experimentally. In addition, relation between expectation and satisfaction should be investigated in future research.
